# Molecular functional analyses revealed essential roles of HSP90 and lamin A/C in growth, migration, and self-aggregation of dermal papilla cells

**DOI:** 10.1038/s41420-018-0053-6

**Published:** 2018-05-09

**Authors:** Kanchalit Thanomkitti, Kedsarin Fong-ngern, Kanyarat Sueksakit, Rattapon Thuangtong, Visith Thongboonkerd

**Affiliations:** 1 Department of Dermatology, Faculty of Medicine Siriraj Hospital, Mahidol University, Bangkok, Thailand; 2 Medical Proteomics Unit, Office for Research and Development, Faculty of Medicine Siriraj Hospital, Mahidol University, Bangkok, Thailand

## Abstract

Previous expression study using quantitative proteomics has shown that immune-mediated pathway may not be the main mechanism inducing alopecia areata (AA). Nevertheless, functional impact of such expression data set remained unknown and unexplored. This study thus aimed to define potentially novel mechanisms of the AA pathogenesis by functional investigations of the differentially expressed proteins previously identified from lesional biopsies. From 122 altered proteins, protein–protein interactions network analysis revealed that downregulated heat shock protein 90 (HSP90) and lamin A/C served as the central nodes of protein–protein interactions involving in several crucial biological functions, including cytoskeleton organization, extracellular matrix organization, and tissue development. Interaction between HSP90 and lamin A/C in dermal papilla cells (DPCs) was confirmed by reciprocal immunoprecipitation and immunofluorescence co-staining. Small-interfering RNA (siRNA) targeting to HSP90 (siHSP90) and lamin A/C (siLamin A/C) effectively reduced levels of HSP90 and lamin A/C, respectively and vice versa, comparing to non-transfected and siControl-transfected cells, strengthening their interactive roles in DPCs. Functional investigations revealed that DPCs transfected with siHSP90 and siLamin A/C had defective cell proliferation and growth, prolonged doubling time, cell cycle arrest at G0/G1 phase, and defective self-aggregation formation. Moreover, siHSP90-transfected cells had less spindle index, reduced levels of vimentin (mesenchymal marker) and fibronectin (extracellular matrix), and defective migratory activity. Our data have demonstrated for the first time that HSP90 and lamin A/C physically interact with each other. Moreover, both of them are essential for growth, migration, and self-aggregation of DPCs and can be linked to the disease mechanisms of AA.

## Introduction

Based on histopathology that frequently shows inflammatory lymphocytes around hair follicles and on clinical response after immunosuppressive treatment, autoimmune mechanism has been hypothesized to play a major role in the disease pathogenesis of alopecia areata (AA)^1^. In a murine model, the data has shown that localized hair loss may be mediated mainly by CD8^+^ T lymphocytes, whereas CD4^+^ T cells can further drive the immune system resulting to the development of multiple AA^2^. Another autoimmune mechanism that may be associated with AA is the collapse of immune privilege of hair follicles due to specific autoantigens, particularly melanogenesis-associated peptides^3^. Moreover, genetic susceptibility associated with specific alleles of both HLA and non-HLA regions may be also related to AA disease mechanisms^4,5^.

Nevertheless, our previous unbiased proteomics study of lesional vs. non-lesional biopsies taken from AA patients has shown that immune-mediated pathway may not be the main mechanism inducing AA pathogenesis^6^. In contrast, most of the differentially expressed proteins identified from this study seem to get involved in potentially novel mechanisms of AA^6^. Unfortunately, functional significance and impact of such large-scale expression data set remained unknown and unexplored. Our present study thus aimed to define potentially novel mechanisms of the AA pathogenesis by functional investigations of such large-scale expression proteomics data set. All the differentially expressed proteins identified from lesional vs. non-lesional biopsies were subjected to global protein–protein interactions network analysis. The central nodes of such protein–protein interactions network were then validated by reciprocal immunoprecipitation and immunofluorescence co-staining, and then subjected to protein knockdown using small-interfering RNA (siRNA) in dermal papilla cells (DPCs) (which are the major compositions of hair follicles and are crucial for hair growth) followed by functional investigations of various cellular functions that might be linked to the pathogenic mechanisms of AA.

## Results

### Global protein–protein interactions network analysis

**Fig. 1:**
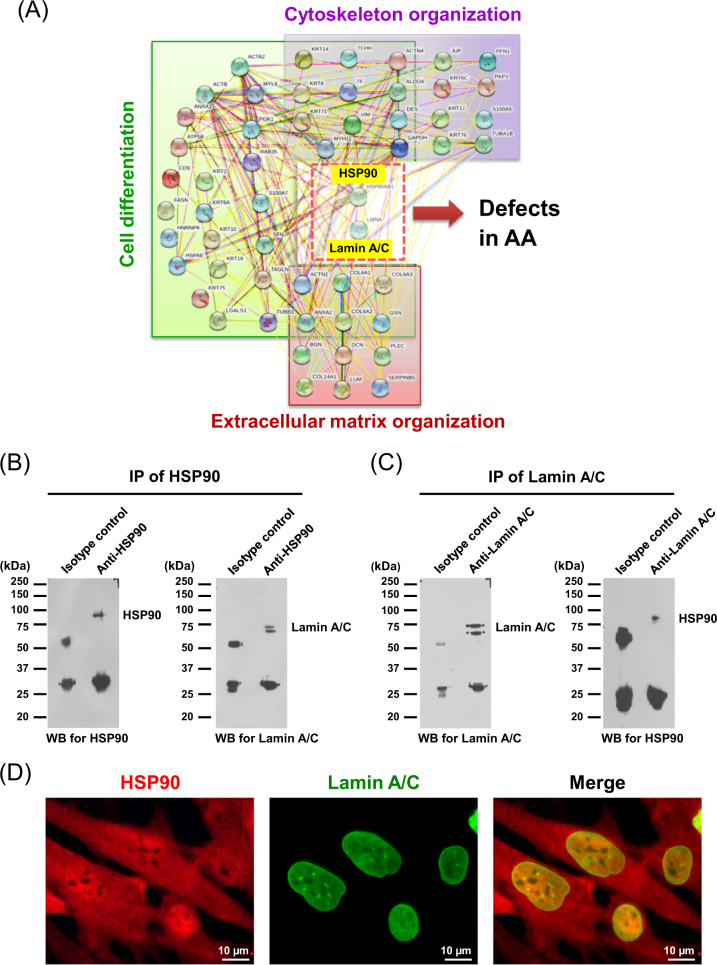
**Protein–protein interactions and significant roles of HSP90 and lamin A/C.** **a** Global protein–protein interactions network analysis revealed significant roles of HSP90 and lamin A/C in cytoskeleton organization, extracellular matrix organization, and tissue development. **b** Reciprocal immunoprecipitation revealed physical interactions between HSP90 and lamin A/C. **c** Immunofluorescence co-staining confirmed their co-localization at perinuclear region (original magnification = ×1000 in all panels). IP immunoprecipitation, WB Western blotting

Global protein–protein interactions network analysis of all differentially expressed proteins in lesional vs. non-lesional biopsies of AA patients identified from our previous proteomics study^6^ revealed that heat shock protein (HSP90) and lamin A/C, both of which were decreased in lesional biopsies^6^, served as the central nodes of protein–protein interactions involving in several crucial biological functions, including cytoskeleton organization, extracellular matrix organization, and tissue development (Fig. 1a). We thus postulated that defects in these two proteins could lead to the pathogenic mechanisms of AA.

### Validation of the interaction between HSP90 and lamin A/C

Because protein–protein interactions networks analysis showed that both central nodes (HSP90 and lamin A/C) of these interactions interacted to each other directly, we thus validated their physical interaction experimentally in DPCs, which are the major cells in hair follicles. Reciprocal immunoprecipitation reveled that lamin A/C was present in the protein complex immunoprecipitated with anti-HSP90 antibody (Fig. 1b) and, vice versa, HSP90 was also present in that immunoprecipitated with anti-lamin A/C antibody (Fig. 1c). Moreover, immunofluorescence co-staining confirmed co-localization of these two proteins in the perinuclear region of DPCs (Fig. 1d).

### Functional analyses of HSP90 and lamin A/C by small-interfering RNA-mediated protein knockdown in DPCs

**Fig. 2:**
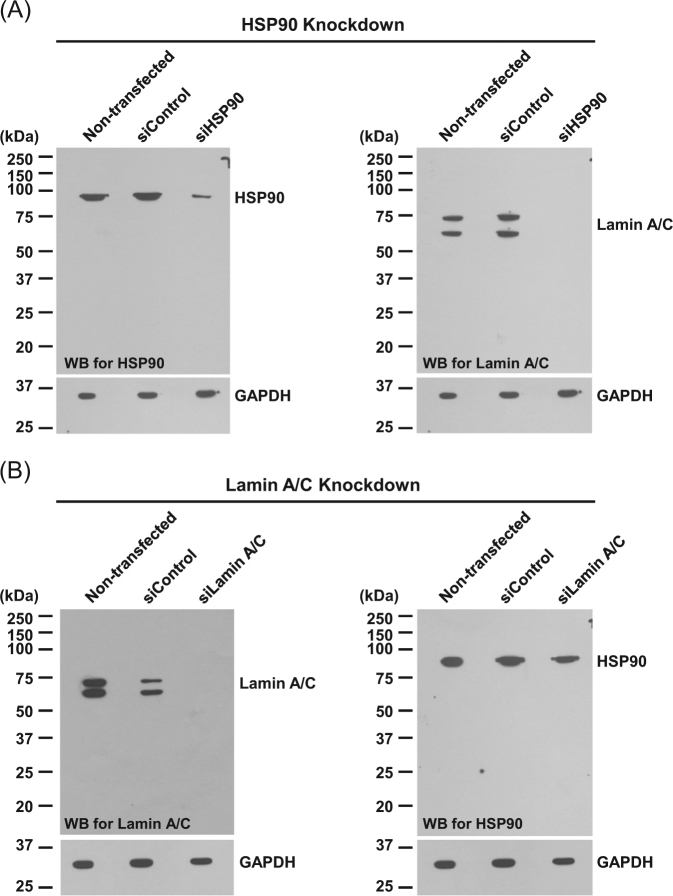
**siRNA-mediated knockdown of HSP90 and lamin A/C in DPCs.** **a** Western blot analysis showed decreased levels of HSP90 and lamin A/C in DPCs transfected with siRNA targeting on HSP90 (siHSP90) as compared to non-transfected and siControl-transfected cells. **b** Western blot analysis showed decreased levels of lamin A/C and HSP90 in DPCs transfected with siRNA targeting on lamin A/C (siLamin A/C) comparing to non-transfected and siControl-transfected cells. GAPDH served as the loading control. WB Western blotting

The efficacies of siRNA targeting to HSP90 (siHSP90) and lamin A/C (siLamin A/C) were then confirmed by Western blotting, which showed markedly decreased levels of both proteins by their corresponding siRNAs (Fig. 2a, b). Additionally, siHSP90 and siLamin A/C also effectively reduced levels of lamin A/C and HSP90, respectively (Fig. 2a, b), strengthening their interactive roles in DPCs.

**Fig. 3:**
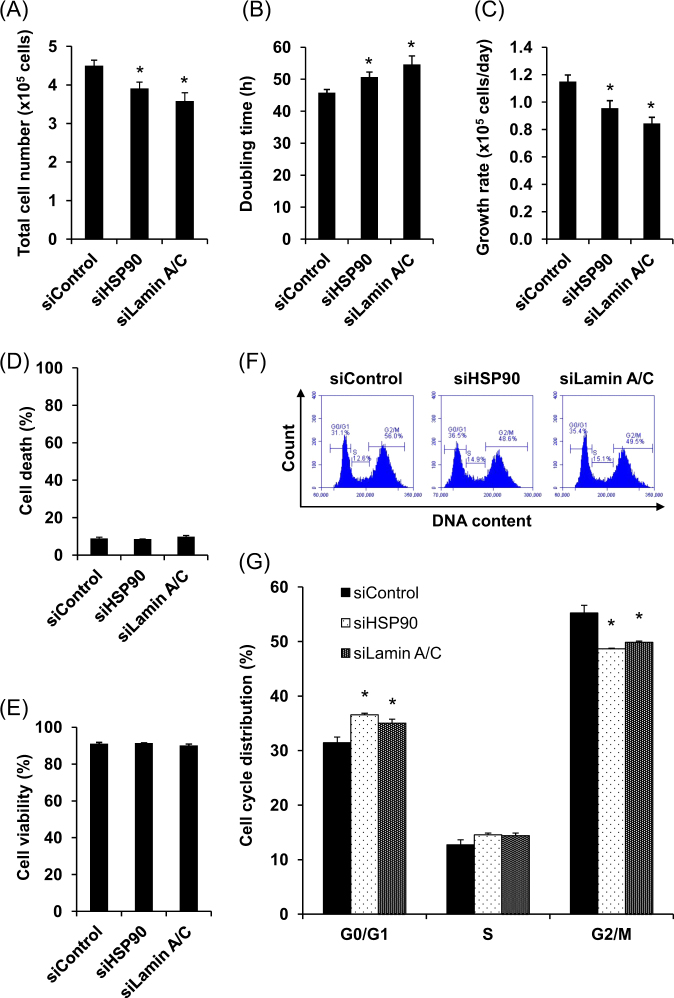
**Effect of siHSP90 and siLamin A/C on DPCs cell growth, viability and cell cycle.** DPCs transfected with siControl, siHSP90 or siLamin A/C for 72 h were detached and counted to determine total cell number (**a**), doubling time (**b**), growth rate (**c**), cell death (**d**), and viability (**e**). Cell cycle distribution was evaluated by staining DNA content using propidium iodide followed by flow cytometry. DNA content distribution pattern in each sample group was plotted as the histogram (**f**) and percentage of the cells in each phase of cell cycle (G0/G1, S, and G2/M) was quantitated (**g**). Each bar represents mean ± SEM of the data obtained from three independent experiments. *p < 0.05 vs. siControl

Effects of siHSP90 and siLamin A/C on various cellular functions of DPCs were then examined comparing to the siControl-transfected cells. The data showed that siHSP90 and siLamin A/C significantly decreased total cell number and growth rate, and concordantly prolonged cell doubling time (Fig. 3a–c). However, both siRNAs did not affect cell death and viability (Fig. 3d, e), indicating that the cytotoxicity from siRNAs could be excluded. Cell cycle analysis by flow cytometry revealed that both siHSP90 and siLamin A/C caused cell cycle shift from G2/M phase to arrest at G0/G1 phase (Fig. 3f, g).

### Effect of siHSP90 and siLamin A/C on spindle morphology, mesenchymal phenotypes, and extracellular matrix production of DPCs

**Fig. 4:**
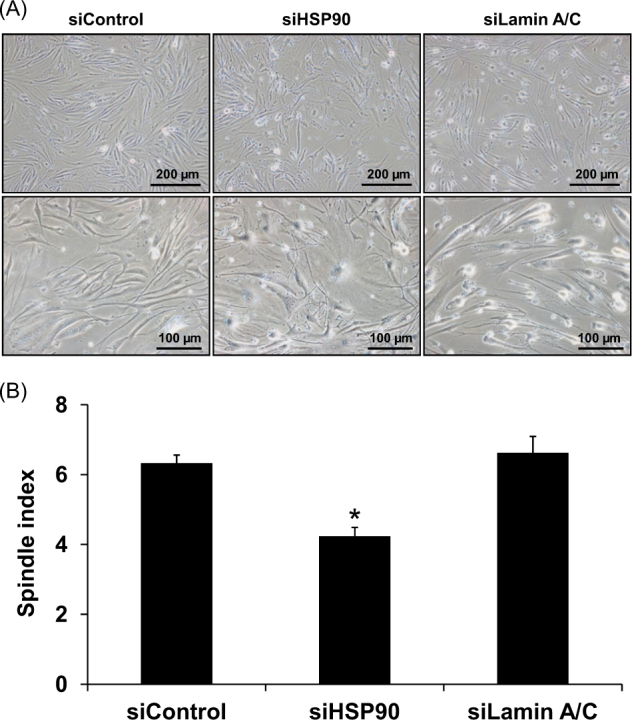
**Effect of siHSP90 and siLamin A/C on spindle morphology of DPCs.** **a** After transfection for 72 h, the siControl-, siHSP90-, and siLamin A/C-transfected DPCs were observed under a phase contrast microscope (original magnification = ×100 in upper panels and ×200 in lower panels). **b** Spindle indices of individual transfected cells were then calculated from at least 100 cells per sample. Each bar represents mean ± SEM of the data obtained from three independent experiments. *p < 0.05 vs. siControl

**Fig. 5:**
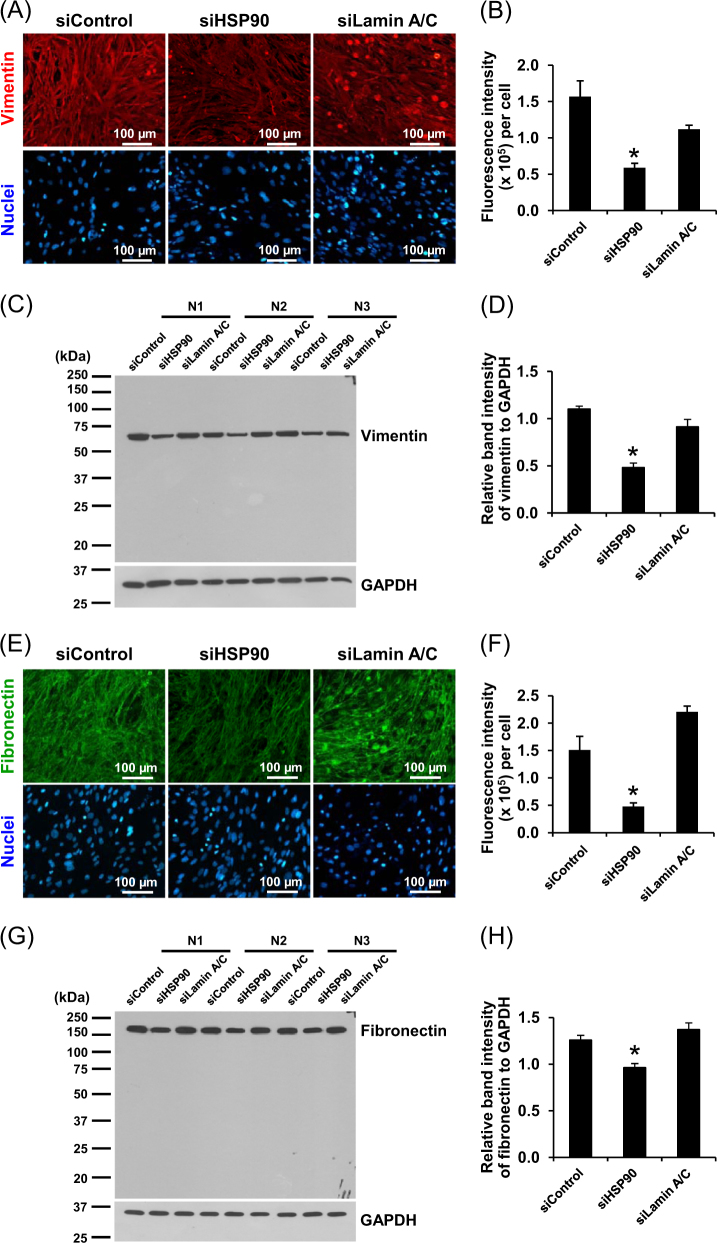
**Effect of siHSP90 and siLamin A/C on mesenchymal phenotypes and extracellular matrix production of DPCs.** **a**,**e** Immunofluorescence staining of mesenchymal marker (vimentin; in red) and extracellular metric protein (fibronectin; in green), respectively. The nuclei were counterstained with Hoechst dye (in blue) (original magnification = ×200 in all panels). **b**,**f** Mean fluorescence intensities representing vimentin and fibronectin levels, respectively, were analyzed from at least 100 cells per sample using NIS-Elements D V.4.11 (Nikon). **c**,**g** Western blot analysis of vimentin and fibronectin, respectively. GAPDH served as the loading control. **d**,**h** Band intensities of vimentin and fibronectin, respectively, were quantitated by using ImageMaster 2D Platinum version 6.0 (GE Healthcare) and were normalized with that of GAPDH. Each bar represents mean ± SEM of the data obtained from three independent experiments. *p < 0.05 vs. siControl

siControl-, siHSP90-, and siLamin A/C-transfected DPCs were examined under a phase contrast microscope. The data showed that suppression of HSP90 by siRNA-affected morphology of DPCs to be less spindle, whereas siLamin A/C seemed to have no effect on such spindle morphology (Fig. 4a). Calculation of spindle index to quantitatively evaluate the spindle morphology of DPCs revealed that siHSP90 significantly decreased the spindle index, whereas siLamin A/C did not change such index (Fig. 4b). In addition to the decreased spindle phenotype, the siHSP90-transfected DPCs showed dendritic morphology instead of their regular shape. Moreover, immunofluorescence staining showed that expression levels of vimentin representing mesenchymal markers^7,8^ (Fig. 5a, b) and fibronectin representing both mesenchymal markers and extracellular matrix production^7,8^ (Fig. 5e, f) were significantly reduced in the siHSP90-transfected DPCs but had no significant changes in the siLamin A/C-transfected cells. In consistent, Western blotting revealed the same findings (Fig. 5c, d, g, and h).

### Effect of siHSP90 and siLamin A/C on migratory activity of DPCs

**Fig. 6:**
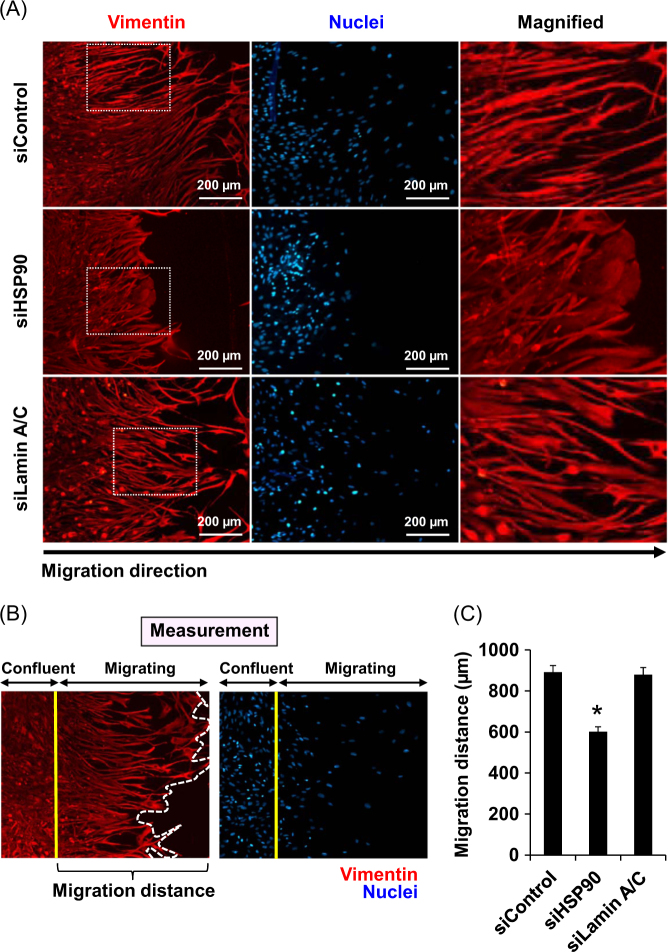
**Effect of siHSP90 and siLamin A/C on migratory activity of DPCs.** **a** After transfection for 72 h, a horizontally scratched line was generated by using a 200-μl plastic pipette tip to create a cell-free area along the culture well diameter of each condition. After further incubation for 24 h, the cells were then subjected to immunofluorescence staining using mouse monoclonal anti-vimentin (in red), whereas the nuclei were counterstained with Hoechst dye (in blue) (original magnification = ×200). Cropped areas were magnified and are shown at the right column. **b** Zoom-in image of the migratory area to illustrate measurement of the migration distance. **c** The migration distance of the cells in each condition was then measured from at least 10 positions per well using Tarosoft® Image framework version 0.9.6 (Nikon). Each bar represents mean ± SEM of the data obtained from three independent experiments. *p < 0.05 vs. siControl

Because siHSP90 affected the spindle morphology and mesenchymal markers of DPCs, we then examined whether siHSP90 also caused functional deteriorations of the cells, particularly cell migration, which is one of the most important features of DPCs to form hair follicles. Using a scratch assay, the data revealed that the siHSP90-transfected cells had much lower migratory activity as compared to the siControl- and siLamin A/C-transfected cells, whereas siLamin A/C did not affect this cellular function (Fig. 6).

### Effect of siHSP90 and siLamin A/C on tissue development as determined by self-aggregation formation of DPCs

**Fig. 7:**
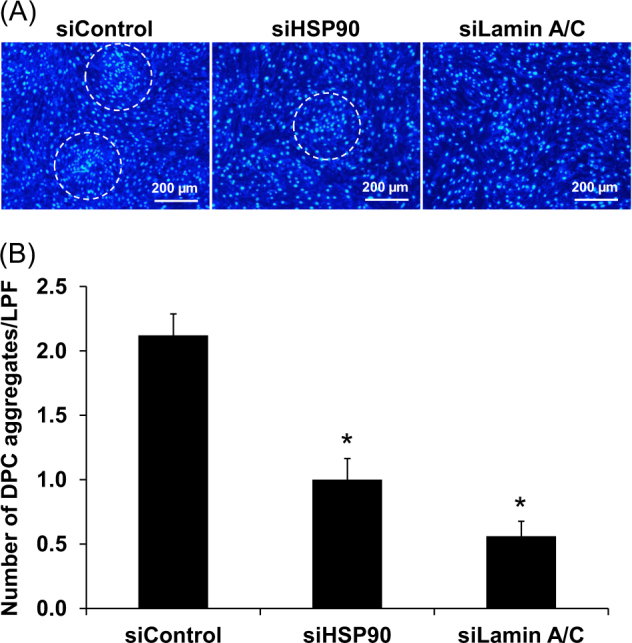
**Effect of siHSP90 and siLamin A/C on self-aggregation formation of DPCs.** **a** The siControl-, siHSP90-, and siLamin A/C-transfected DPCs were seeded at a high-density of 3 × 10^5^ cells in each of 6-well plate and maintained for 72 h to allow self-aggregation formation. Thereafter, the cell nuclei were stained by Hoechst dye (in blue) and the cells were examined under an ECLIPSE 80i fluorescence microscope (Nikon) (Original magnification = ×100). The DPCs’ cell clumps indicating self-aggregation formation are labeled with white dotted circle. **b** Number of the cell aggregates in each condition was counted from at least 25 low-power fields (LPF) per well. Each bar represents mean ± SEM of the data obtained from three independent experiments. *p < 0.05 vs. siControl

DPCs normally have aggregative characteristics both in vivo and in vitro. This self-aggregation ability is crucial for DPCs, together with other cell types and components, to form the hair follicles. We therefore evaluated the effect of siHSP90 and siLamin A/C on self-aggregation formation of DPCs. The data showed that both siHSP90- and siLamin A/C-transfected DPCs had markedly decreased ability to form the cell aggregates as compared to the siControl-transfected cells (Fig. 7). This result implicated that both HSP90 and lamin A/C are crucial for the formation of hair follicles.

## Discussion

AA is one of the common diseases worldwide. Although spontaneous remission within 1 year occur in up to 50% of the patients, recurrent episodes are quite common thereafter^9^. Thus, most of the patients require treatment to gain their normal hair condition. Currently, there are various therapeutic regimens for AA, including intralesional/topical administration of corticosteroids, minoxidil or anthralin, topical immunotherapy (e.g., dinitrochlorobenzene, squaric acid dibutylester, diphenylcyclopropenone), phototherapy, and systemic administration of corticosteroids, steroid-sparing immunosuppressants or biologic agents^10^. However, a large proportion of the patients are refractory to the treatment and/or experience frequent relapsing episodes after hair regrowth^11^. From this unfavorable outcome, together with the clues from our previous proteomics work indicating that immune-mediated pathway may not serve as the major mechanism inducing AA^6^, it is thus most likely that there are still unknown/unexplored mechanisms involving in the pathogenesis of AA.

From the protein–protein interactions network prediction, HSP90 and lamin A/C were involved in cytoskeleton organization, extracellular matrix organization, and tissue development (Fig. 1a). Their physical interaction in DPCs was nicely confirmed by reciprocal immunoprecipitation and immunofluorescence co-staining (Fig. 1b, c). To our knowledge, this is the first direct evidence demonstrating the actual physical interaction between HSP90 and lamin A/C. We then knocked these two proteins down using siRNA technique and examined effects of HSP90- and lamin A/C-knockdown (which simulated their decreased levels in the AA lesional biopsies) in hair follicle-derived cells with regard of cytoskeleton organization, extracellular matrix organization, and tissue development. DPCs, the specialized mesenchymal-derived cells that contain hair regenerating potency and have been extensively used for studying mechanisms of baldness^12^, were employed for such functional investigations.

HSP90 is a chaperone protein that can protect cells and tissues from various stresses and is involved in tissue repairing^13^. Recently, HSP90 had been discovered to be secreted from human keratinocyte (HaCaT) and promoted wound healing and re-epithelialization^14^. In our present study, siHSP90-transfected DPCs showed cellular morphological changes by decreasing their spindle morphology and had more dendritic appearance (Fig. 4). Knockdown of HSP90 also reduced mesenchymal markers, including vimentin and fibronectin, implicating that HSP90 is important for maintenance of mesenchymal characteristics of DPCs. Our findings were consistent with those reported previously indicating that HSP90 involves in the induction and maintenance of epithelial-to-mesenchymal transition of epithelial and cancerous cells^15,16^. Furthermore, we found the reduction of migratory activity of DPCs after HSP90 knockdown (Fig. 6), consistent with the previous findings reporting that HSP90 is involved in migration ability of rat mesenchymal stem cells, cancer cells, keratinocytes, and several other cell types^16–19^. To our knowledge, our data set is the first to demonstrate the relationship between HSP90 and DPCs function, particularly in AA.

Lamin A/C is a major component of the nuclear lamina and has been proposed to play important role in morphogenesis of dermis and subcutaneous tissue^20^. Mutations of *LMNA* gene encoding lamin A/C are associated with a premature aging syndrome (Hutchinson–Gilford syndrome or progeria) that is generally presented with the subcutaneous tissue loss, leg ulceration, joint abnormalities, coronary atherosclerosis and alopecia^21,22^. Our data showed that lamin A/C-knockdown DPCs had defective proliferation and altered cell cycle distribution (Fig. 3). These findings were in concordance with those reported previously on other cell types, suggesting that expression of lamin A/C is influential for the proliferative ability of those various cells and phosphorylation of lamins play critical roles throughout the cell cycle progression^23–25^.

The self-aggregation property of DPCs is essential for induction of hair follicle formation^26^. Our present study showed that both HSP90 and lamin A/C were important for DPCs to form cell clumps (Fig. 7) that, in concert with other cell types and components, can lead to hair follicle formation. Taken together, HSP90 and lamin A/C serve as the essential proteins that are crucial for hair follicle generation and maintenance, whereas their defects may contribute to the pathogenesis of AA via DPCs’ dysfunctions.

In summary, we report herein for the first time that HSP90 and lamin A/C physically interact with each other. Moreover, both of them are essential for growth, migration, and self-aggregation of DPCs and can be linked to the disease mechanisms of AA.

## Materials and methods

### Global protein–protein interactions network analysis

All differentially expressed proteins in lesional vs. non-lesional biopsies of AA patients identified from our previous proteomics study^6^ were subjected to global protein–protein interactions network analysis using Search Tool for the Retrieval of Interacting Genes/Proteins (STRING) version 10.0 (http://string.embl.de). This tool generates protein–protein interaction networks base on integration of both physical and functional linking data from databases of experimental repositories, computational predictions, and public available literatures^27^. Protein interactions and functional connectivity were used for designing subsequent functional investigations.

### Cultivation of dermal papilla cells

DPCs, derived from a human DPC line (ATCC), were cultivated on collagen type I-coated tissue culture flask in Dulbecco’s Modified Eagle Medium/Nutrient Mixture F-12 (DMEM/F12) (GIBCO^TM^, Invitrogen Corporation, Grand Island, NY) supplemented with 10% heat-inactivated fetal bovine serum (FBS), 60 U/ml of penicillin G, 60 µg/ml of streptomycin (Sigma, St. Louis, MO). The cells were maintained in a humidified incubator with 5% CO_2_ at 37 °C. For collagen type I-coated tissue culture flask preparation, purified bovine collagen type I stock solution (3 mg/ml) (PureCol, Advanced Biomatrix, San Diego, CA) was diluted 1:30 in PBS and then added into the culture flask (Corning Costar, Cambridge, MA) just to cover the whole surface area and then incubated at 4 °C overnight. The collagen-coated flask was rinsed twice with PBS before use.

### Reciprocal immunoprecipitation

DPCs were lyzed in a modified RIPA buffer (50 mM Tris-HCl (pH 7.4), 150 mM NaCl, 0.5% Triton X-100, and 1 mM EDTA) and further homogenized by sonication. Cell debris and particulate matters were removed by centrifugation at 10,000 × *g* and 4 °C for 15 min. Protein concentration was measured by Bio-Rad Protein Assay (Bio-Rad Laboratories, Hercules, CA) based on the Bradford’s method. Prior to immunoprecipitation, 2 mg of cell lysate were pre-cleared with 50 µl of protein G sepharose beads (50% slurry) (GE Healthcare, Uppsala, Sweden) at 4 °C on a rotary device for 15 min. Beads with non-specifically bounded proteins were removed by centrifugation at 1500 × *g* and 4 °C for 5 min^28,29^. Thereafter, the sample was incubated with 1 µg of rabbit polyclonal anti-HSP90 antibody (Santa Cruz Biotechnology, Santa Cruz, CA), mouse monoclonal anti-lamin A/C (Santa Cruz Biotechnology), or isotype-controlled rabbit or mouse IgG (Santa Cruz Biotechnology) overnight at 4 °C on a rotary device. Protein G sepharose beads (50 µl) were then added and incubated with each mixture at 4 °C for 4 h. Thereafter, the beads were collected by centrifugation at 1500 × *g* and 4 °C for 5 min and washed three times with 1 ml modified RIPA buffer. The immunoprecipitated proteins were finally eluted from the beads using Laemmli’s buffer and subjected to Western blotting as detailed below.

### Western blotting

For immunoprecipitated samples, proteins eluted from the beads were resolved in each lane of 12% SDS-PAGE gel. For non-immunoprecipitated samples, the cells were harvested by direct scraping into an Eppendorf tube. After washing with PBS, the cells were incubated with Laemmli’s buffer at 25 °C for 30 min. The supernatant was collected after centrifugation at 10,000 × *g* and 4 °C for 30 min and protein concentration was measured by Bio-Rad Protein Assay based on the Bradford’s method. Equal amount of total protein (30 µg/lane) from each sample was resolved by 12% SDS-PAGE.

The resolved proteins were then transferred onto a nitrocellulose membrane. After blocking non-specific bindings with 5% skim milk in PBS for 1 h, the membrane was incubated with mouse monoclonal anti-HSP90, anti-lamin A/C, anti-vimentin, anti-fibronectin, or anti-GAPDH antibody (all were purchased from Santa Cruz Biotechnology and diluted 1:1000 in 1% skim milk in PBS) at 4 °C overnight. After probing with corresponding secondary antibody conjugated with horseradish peroxidase at a dilution of 1:2000 in 1% skim milk in PBS at 25 °C for 1 h, the immunoreactive protein bands were visualized by SuperSignal West Pico chemiluminescence substrate (Pierce Biotechnology, Inc., Rockford, IL) and autoradiography. Band intensity data was obtained using ImageMaster 2D Platinum version 6.0 (GE Healthcare)^30,31^.

### Immunofluorescence staining and co-staining

The non-transfected, siControl-transfected, siHSP90-transfected, and siLamin A/C-transfected DPCs were grown in each of 6-well plate at a density of 7.5 × 10^4^ cells for 72 h. The cells were fixed with 4% paraformaldehyde for 15 min and subsequently permeabilized by 0.1% TritonX-100 in PBS for 15 min at 25 °C. For single staining, the cells were incubated with mouse monoclonal anti-vimentin antibody or mouse monoclonal anti-fibronectin (both were from Santa Cruz Biotechnology and diluted 1:50 in 1% BSA/PBS) at 37 °C for 1 h. For co-staining, the cells were incubated with both rabbit polyclonal anti-HSP90 antibody and mouse monoclonal anti-lamin A/C (both were from Santa Cruz Biotechnology and diluted 1:50 in 1% BSA/PBS) at 37 °C for 1 h. The cells were rinsed with PBS three times and further incubated with Alexa Flour^®^ 555-conjugated or Alexa Fluor^®^ 488-conjugated secondary antibody (both were from Invitrogen-Molecular Probes, Burlinton, ON, Canada; and were diluted 1:2000 in 1%BSA/PBS) mixed with 0.1 µg/ml Hoechst dye (DNA staining for nuclear localization) (Sigma, St. Louis, MO) at 37 °C for 1 h. Thereafter, the stained cells were washed with PBS and mounted with 50% glycerol/PBS for subsequent examination using an ECLIPSE 80i fluorescence microscope (Nikon). Mean fluorescence intensity representing protein level was analyzed from 10 random high-power fields (at least 100 cells) in each well using NIS-Elements D V.4.11 (Nikon)^32,33^.

### Transient knockdown of HSP90 and lamin A/C by small-interfering RNA

DPCs were grown in 6-well or 24-well plate in antibiotic-free DMEM/F12 supplemented with 10% FBS for 18–24 h to obtain 60–80% confluency. siRNA targeting to HSP90 (siHSP90) (Santa Cruz Biotechnology) or lamin A/C (siLamin A/C) (Santa Cruz Biotechnology) was transfected to the cells using a commercially available transfection kit (Santa Cruz Biotechnology) following the manufacturer’s instructions. Briefly, DPCs were incubated with 50 pmol of siHSP90 or siLamin A/C, which were mixed with siRNA transfection reagent in siRNA transfection medium (Santa Cruz Biotechnology). The controlled siRNA (siControl), at an equal dosage, served as the control of this transfection system. The transfected cells were incubated in a humidified incubator with 5% CO_2_ at 37 °C for 5 h and then refreshed with DMEM/F12 supplemented with 10% FBS for additional 72 h before subsequent confirmation and functional investigations.

### Total cell count and quantitative analysis of cell death, viability, doubling time, and growth rate

The siControl-, siHSP90-, and siLamin A/C-transfected DPCs were seeded at a density of 7.5 × 10^4^ cells in each well of 6-well plate for 72 h. All the transfected cells were detached with 0.05% trypsin/0.02% EDTA in Hank’s balanced salt solution (HBSS) and then stained with trypan blue. Total number of the cells and numbers of dead and viable cells were counted under a phase contrast microscope (Olympus CKX41, Olympus Co. Ltd., Tokyo, Japan) using a hemacytometer. Doubling time and growth rate of the cells were calculated using the formulas^34^:

$${\rm{Formula}}\;1:\;{\rm{Doubling}}\;{\rm {time}}\;{\left( h \right)} = {\left( {\rm{ln}}\;{2t} \right)}/{\left( {\rm{ln}}\;{X_{\rm{t}}/X_0} \right)}$$

$${\mathrm{Note}}:{t} = {\mathrm{the}}\;{\mathrm{incubation}}\;{\mathrm{time}}\;{\left( {72\;{\mathrm{h}}\;{\mathrm{in}}\;{\mathrm{this}}\;{\mathrm{study}}} \right)}$$

$${X}_{\mathrm{t}} = {\mathrm{total}}\;{\mathrm{cell}}\;{\mathrm{number}}\;{\mathrm{at}}\;72\;{\mathrm{h}}\;{\mathrm{after}}\;{\mathrm{seeding}}$$

$${X}_0 = {\mathrm{initial}}\;{\mathrm{cell}}\;{\mathrm{number}}\;{\mathrm{seeded}}$$

$${\rm{Formula}}\;2:\;{\rm{Growth}}\;{\rm{rate}}\;{\left( {\rm{cells/day}} \right)} \\ = {\rm{Total}}\;{\rm{cell}}\;{\rm{number/Incubation}}\;{\rm{time}}.$$

### Cell cycle analysis

The siControl-, siHSP90-, and siLamin A/C-transfected DPCs were seeded at a density of 1.8 × 10^5^ cells in each of 60-mm tissue culture dish and maintained for 72 h. The cells were collected by trypsinization, fixed, and permeabilized with ice-cold 70% ethanol, and incubated on ice for 30 min. Thereafter, the cells were washed twice and resuspended in PBS containing 100 µg/ml RNase A (Sigma-Aldrich, St. Louis, MO). After incubation for 30 min, the cells were stained with propidium iodide (BD Biosciences, San Jose, CA) at 25 °C in the dark for 10 min. The stained cells were finally analyzed for their DNA content using BD Accuri C6 flow cytometer (BD Accuri, Beckman Coulter, Fullerton, CA). At least 10,000 events per each sample were evaluated and the cell cycle histograms were generated by BD Accuri™ C6 software in order to quantify the percentage of cells in G0/G1, S, and G2/M phases^34,35^.

### Measurement of spindle index of DPCs

The siControl-, siHSP90-, and siLamin A/C-transfected DPCs were grown in each of 6-well plate at a density of 7.5 × 10^4^ cells for 72 h. Cell morphology of each condition was observed and imaged under a phase contrast microscope (Olympus CKX41). Spindle index of each transfected group was calculated from at least 100 cells per well using the following formula.

$${\rm{Formula}}\;3:\;{\rm{Spindle}}\;{\rm{index}} = {\rm{Length}}{\left( {\mu m} \right)}/{\rm{Width}}{\left( {\mu m} \right)},$$

Note: Length and width were measured by using the Tarosoft® Image framework version 0.9.6 (Nikon, Tokyo, Japan).

### Examination of DPCs migration

The siControl-, siHSP90-, and siLamin A/C-transfected DPCs were grown in a 6-well plate at a density of 7.5 × 10^4^ cells/ml for 72 h to obtain confluent monolayer. A horizontally scratched line was generated by using a 200-μl plastic pipette tip to create a cell-free area along the culture well diameter of each condition. After washing with PBS to remove the detached cells and debris, the cells were further maintained at 37 °C with 5% CO_2_ for 24 h to allow cell migration. The migrated cells from all transfected conditions were fixed with 4% paraformaldehyde for 15 min and permeabilized with 0.1% TritonX-100 in PBS for 15 min at 25 °C. The cells were then incubated with anti-vimentin antibody (Santa Cruz Biotechnology) (1:1000 in 1% BSA/PBS) at 37 °C for 1 h, rinsed with PBS three times and further incubated with Alexa Flour^®^ 555-conjugated secondary antibody (Invitrogen-Molecular Probes) (1:2000 in 1%BSA/PBS) at 37 °C for 1 h. Thereafter, the stained cells were washed with PBS and mounted with 50% glycerol/PBS for subsequent imaging using an ECLIPSE 80i fluorescence microscope (Nikon). All images were submitted to Tarosoft^®^ Image framework version 0.9.6 (Nikon) to accurately measure the migratory distance of the cells in each condition from at least 10 positions per well^36,37^.

### Examination of DPCs self-aggregation formation

The siControl-, siHSP90-, and siLamin A/C-transfected DPCs were seeded at a high-density of 3 × 10^5^ cells in a 6-well plate and maintained for 72 h to allow self-aggregation formation. Thereafter, the cells were rinsed with PBS and fixed with 4% paraformaldehyde for 15 min and permeabilized with 0.1% TritonX-100 in PBS for 15 min at 25 °C. After washing three times with PBS, the cells were then incubated with 0.1 µg/ml Hoechst dye in 1% BSA/PBS at 25 °C for 30 min in the dark to stain cellular nuclei. The stained cells were then examined under an ECLIPSE 80i fluorescence microscope (Nikon) and the number of DPCs aggregates in each condition was counted from at least 25 fields per well.

### Statistical analysis

All experiments were done in triplicates unless stated otherwise. Comparisons between the two groups of samples were performed using unpaired Student’s *t*-test, whereas multiple comparisons of more than two groups of samples were performed using one-way analysis of variance (ANOVA) with Tukey’s post hoc test. *P* < 0.05 were considered statistically significant.
